# Supplementary material for: Maresin-1 Prevents Liver Fibrosis by Targeting Nrf2 and NF-κB, Reducing Oxidative Stress and Inflammation
Source: Cells. 2021 Dec 3;10(12):3406. doi: 10.3390/cells10123406 (PMC8699629; doi:10.3390/cells10123406)

Supplementary

S1: liver biopsies analysis

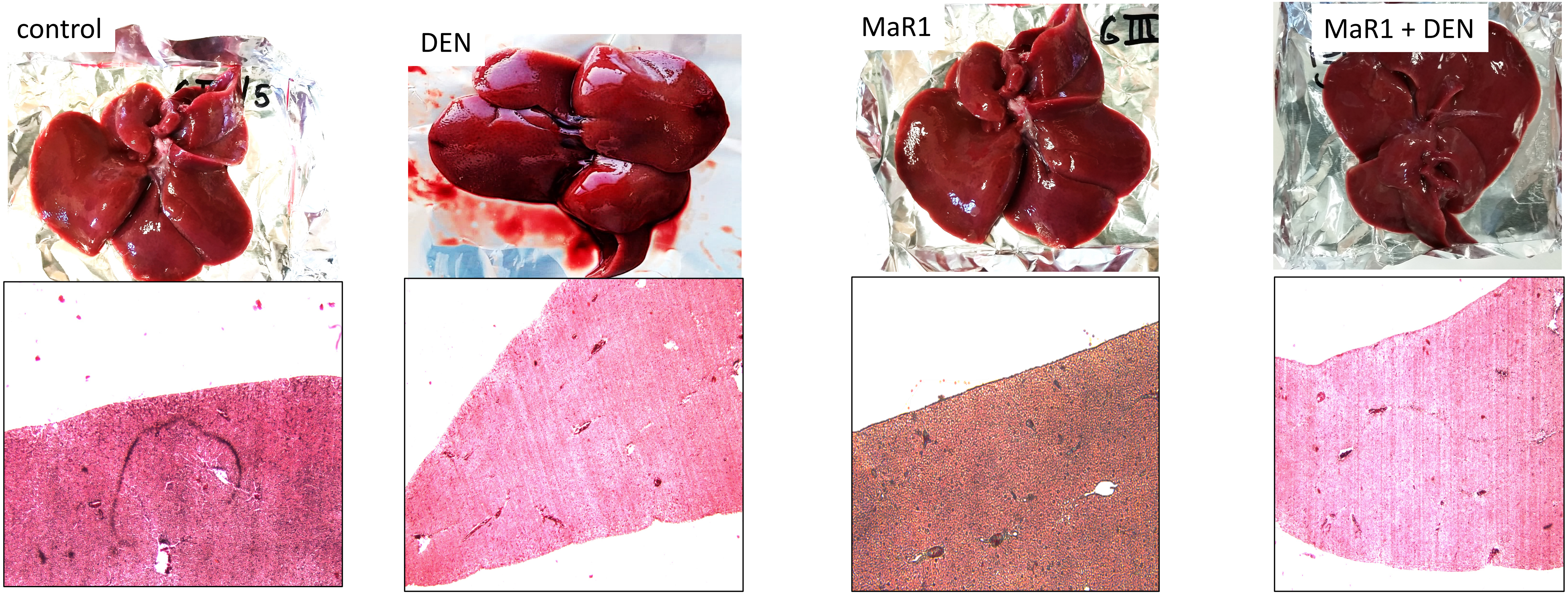

S2: Liver Van Gieson stain

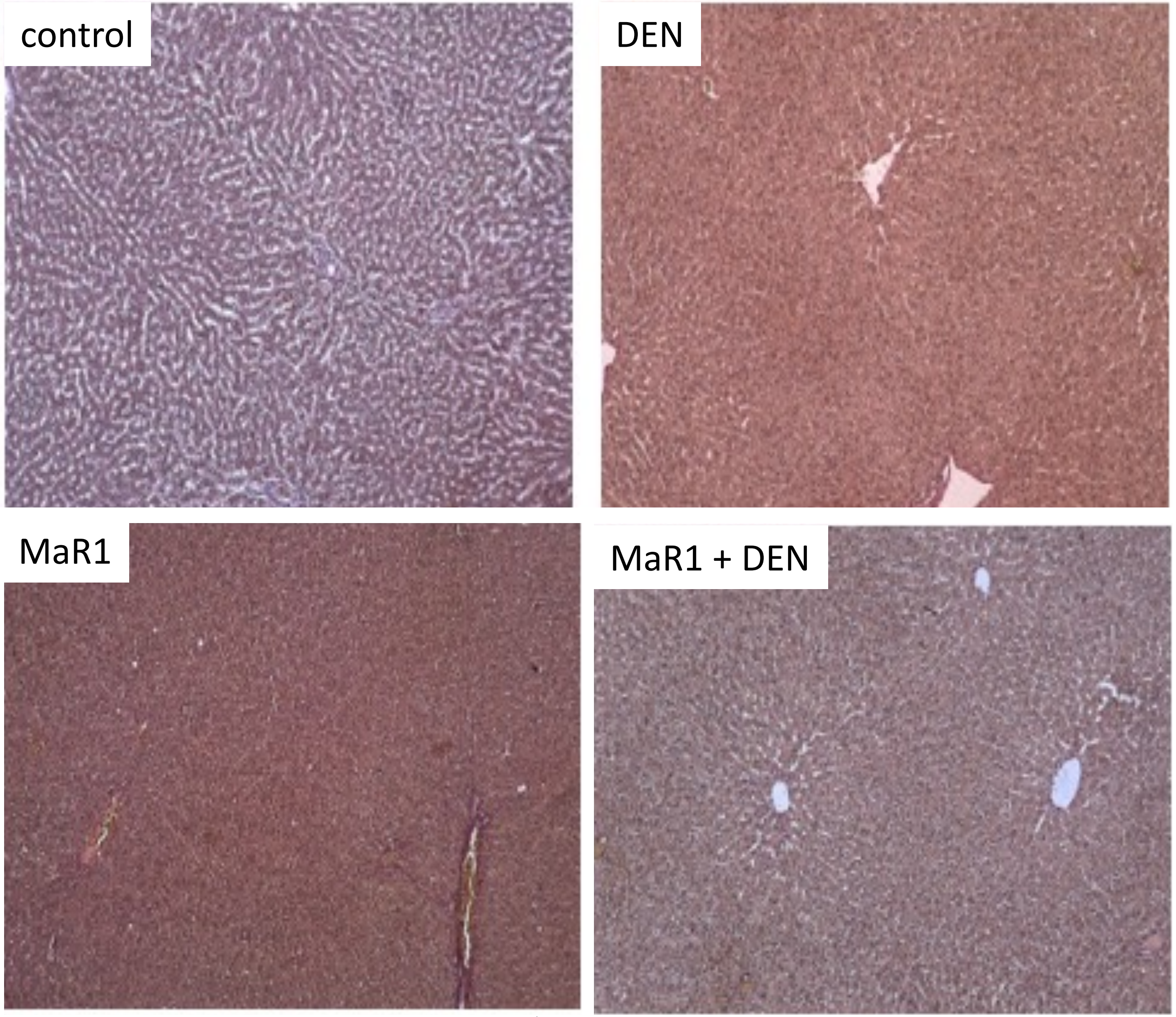

Van Gieson 100x.

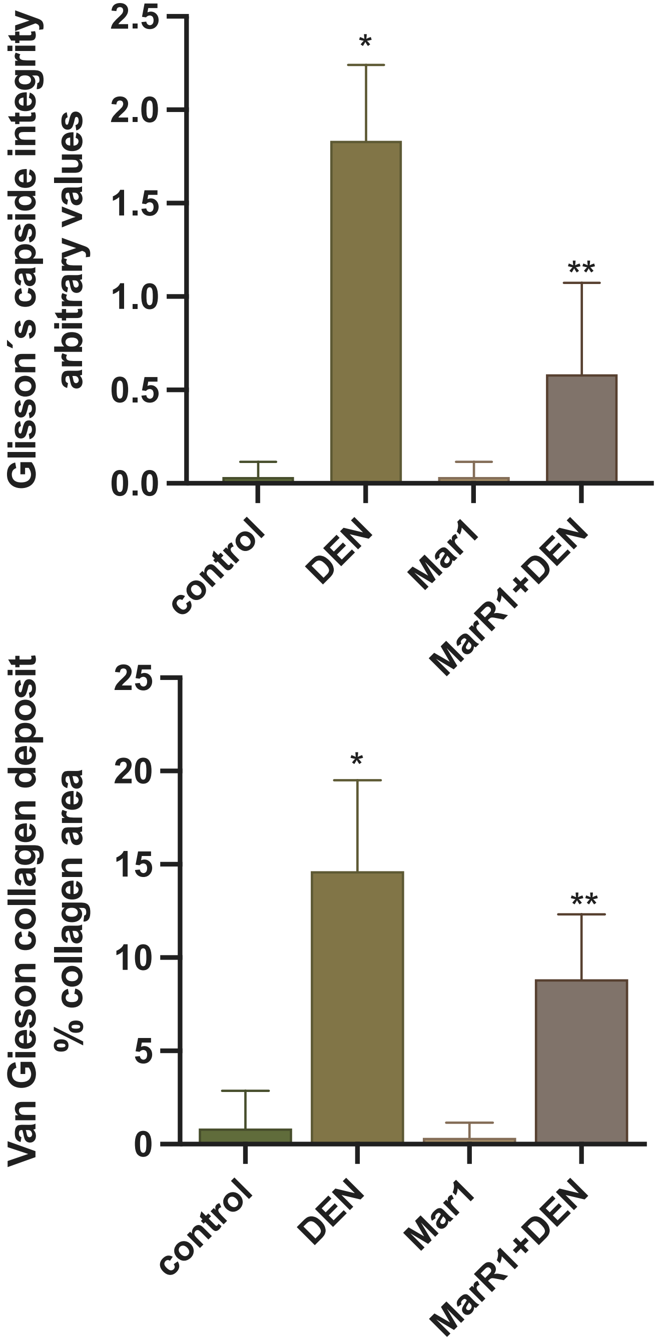

S3: Liver Elastic Van Gieson stain

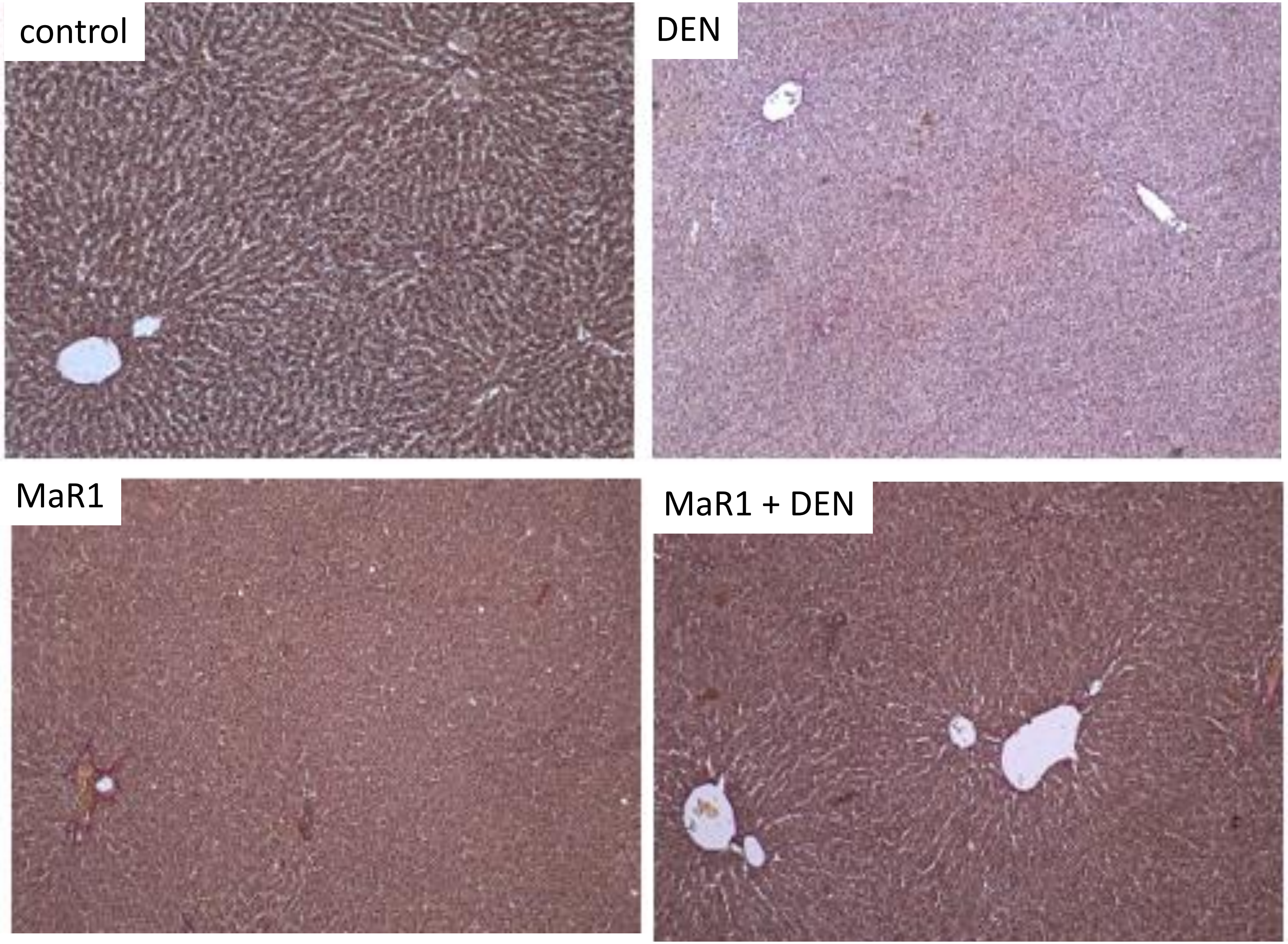

Elastic Van Gieson stain 100x

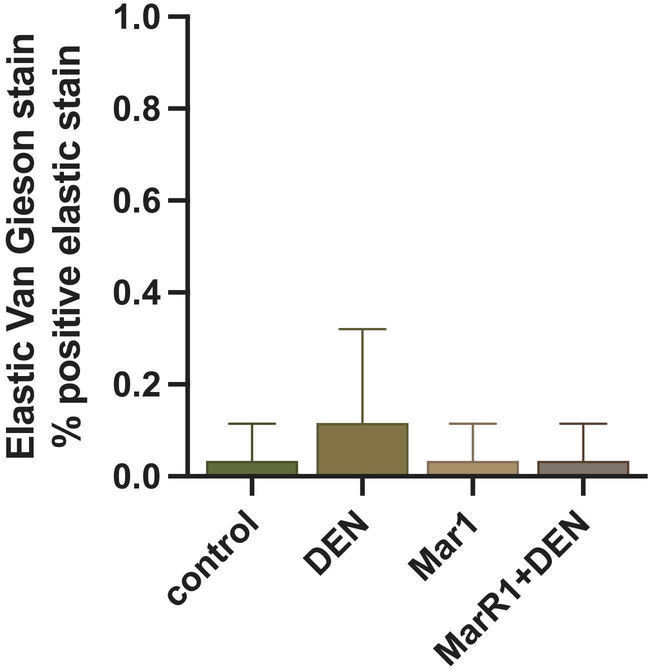

S4: Liver Periodic-ácid Schiff (PAS)

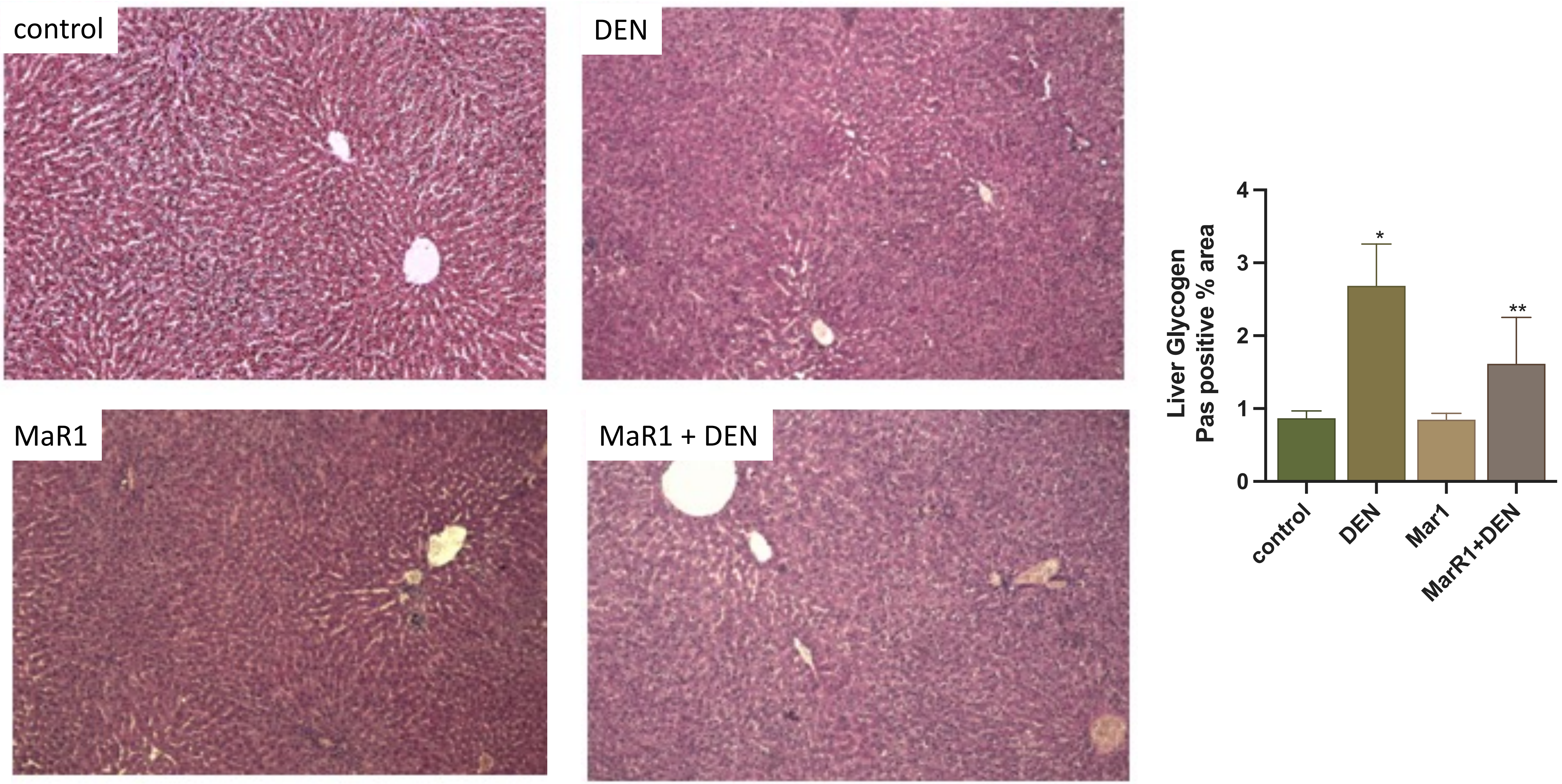

PAS stain 100x

S5: IκBα and *p*-IκBα western blot

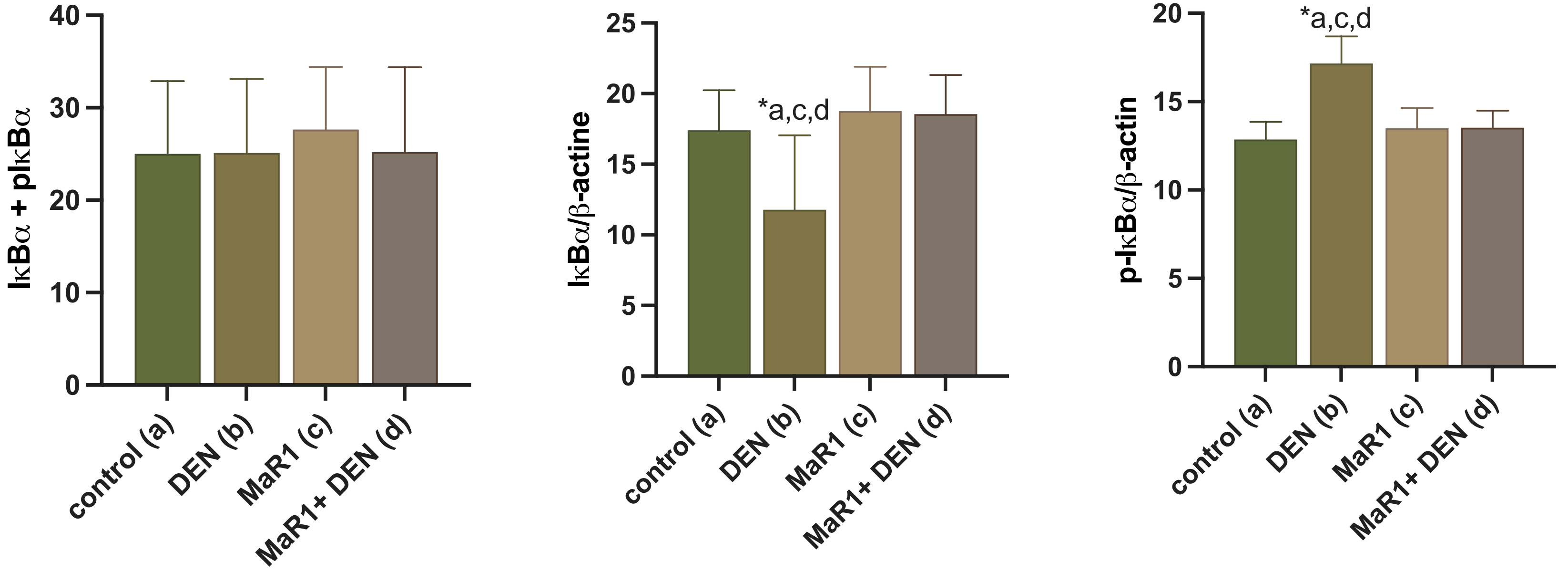

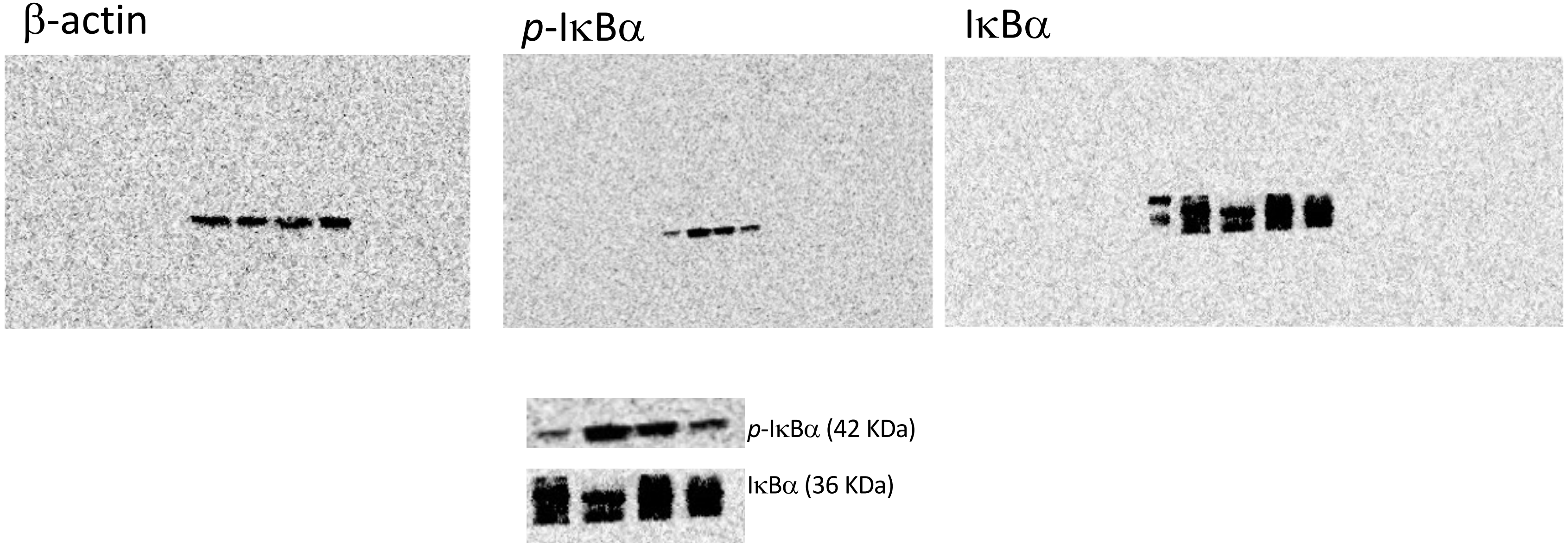

S6: GSH and GSSG liver tissue analysis

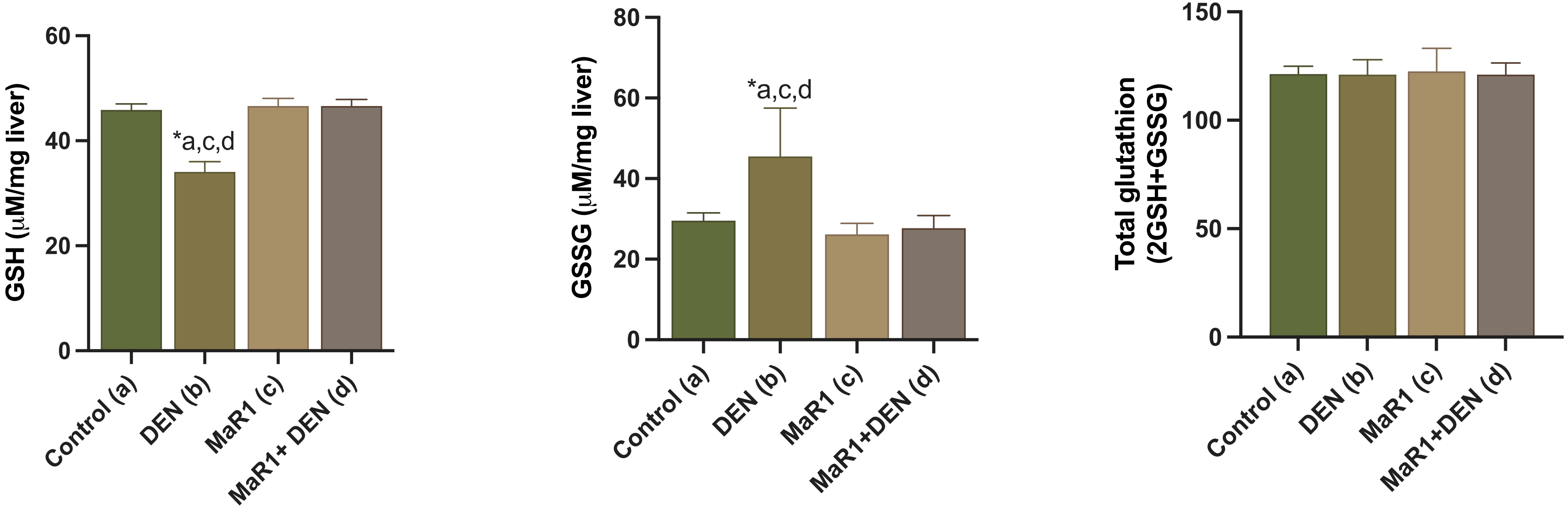

Supplement: Supplementary file 1 [file cells-10-03406-s001.zip › cells-1429221-supplementary.pdf]
